# Experiences and coping with the altered body image in digestive stoma patients**^1^**

**DOI:** 10.1590/1518-8345.1276.2840

**Published:** 2016-12-08

**Authors:** César Hueso-Montoro, Candela Bonill-de-las-Nieves, Miriam Celdrán-Mañas, Sandra Milena Hernández-Zambrano, Manuel Amezcua-Martínez, José Miguel Morales-Asencio

**Affiliations:** 2 PhD, Assistant Professor, Facultad de Ciencias de la Salud, Universidad de Granada, Granada, Spain.; 3 PhD, RN, Servicio Andaluz de Salud, Almería, Spain.; 4 MSc, RN, Servicio Andaluz de Salud, Almería, Spain.; 5 MSc, Researcher, Fundación Pública Andaluza para la Investigación Biosanitaria de Andalucía Oriental - Alejandro Otero, Granada, Spain.; 6 PhD, Associate Professor, Facultad de Ciencias de la Salud, Universidad de Granada, Granada, Spain.; 7 PhD, Professor, Facultad de Ciencias de la Salud, Universidad de Málaga, Málaga, Spain.

**Keywords:** Adaptation, Psychological, Body Image, Colostomy, Ileostomy, Qualitative Research

## Abstract

**Objective:**

to describe the coping of stoma patients with the news about the ostomy, as well as to analyze the meaning and the experience of their new bodily reality.

**Method:**

qualitative phenomenological study undertaken through semistructured interviews with 21 stoma patients. The analysis was based on the constant comparison of the data, the progressive incorporation of subjects and triangulation among researchers and stomal therapy nurses. The software Atlas.ti was used.

**Results:**

two main categories emerge: "Coping with the news about receiving a stoma" and "Meaning and experience of the new bodily reality". The informants' answer varies, showing situations that range from the natural acceptance of the process to resignation and rejection. The previous experiences of other family members, the possible reconstruction of the stoma or the type of illness act as conditioning factors.

**Conclusions:**

the coping with the news about the stoma is conditioned by the type of illness, although the normalization of the process is the trend observed in most informants. Nursing plays a fundamental role in the implementation of cognitive-behavioral interventions and other resources to promote the patients' autonomy in everything related to care for the stoma.

## Introduction

Colorectal cancer (CRC) and Inflammatory Bowel Diseases (IBD) are the conditions that frequently lead to the installation of a digestive stoma. CRC ranks first in terms of incidence level and is the second cause of death in Europe in men and women, with 446,000 new cases being diagnosed each year^1^. 

Stoma patients face different losses at the physical and functional as well as at the psychological, emotional and social level^2^ ^-^ ^7^.

To cope with this situation, people make use of different coping strategies, applying new resources and skills to adapt to their new bodily reality^8^. That is described as the cognitive and behavioral efforts to respond to the specific demands in view of a situation that is perceived as problematic and that target the reestablishment of balance^9^. Based on this premise, the experience of stoma patients should be investigated with a view to understanding how they experience and cope with their new bodily reality, thus constituting a theoretical knowledge base to propose cognitive-behavioral interventions in clinical practice that help them to cope with their new situation. 

In that sense, recent studies suggest that additional research is needed about the aspects that concern stoma patients and that affect their health-related quality of life^10^ ^-^ ^11^. Other studies directly appoint the need for studies that propose a comprehensive concept of bodily change, which allows the health services to better approach these people's individual needs^12^ ^-^ ^13^.

In line with the above, due to the need for in-depth knowledge on the suffering of digestive stoma patients concerning the change in their body image, the objective in this study was to describe how people who are going to receive a stoma cope with the news, as well as to analyze the meaning and the experience of their new bodily reality.

## Method

Descriptive and qualitative study with a phenomenological focus^14^, based on Husserl's descriptive phenomenology. Participants were male and female digestive stoma patients living in Malaga and Granada (Spain). People with a cognitive deficit were excluded. The informants were located through three stomal therapy nurses affiliated with the Hospital Universitario Virgen de la Victoria de Málaga, Hospital Universitario San Cecilio de Granada and Hospital Costa de Sol de Marbella. These hospitals are part of the Public Health System network in Andalucia, Spain.

In the selection of the informants, the researchers tried to guarantee the presence of different profiles surrounding the research problem. The diversification criteria were: illness (Cancer, Crohn's Disease, Ulcerative Colitis, Familial Polyposis), type of digestive stoma (colostomy, ileostomy), dwelling time of the stoma (temporary or permanent) and sociodemographic criteria (age, sex).

The theoretical sampling guidelines were followed. The saturation point was reached at 21 informants. Twelve of them were men and nine women, between 20 and 75 years of age; 14 had a digestive stoma due to an oncologic process, six due to IBD and one to Familial Polyposis. Of all informants, ten had an ileostomy and 11 a colostomy. The dwelling time of the stoma was permanent in 13 cases and temporary in eight.

The data were collected through semistructured interviews that took between 35 and 40 minutes and were audio-recorded. Ten interviews took place at the informants' homes and 11 at the stomal therapy nurse's consultation room, with the help of an interview script. All interviews were held face to face by the same researcher and without the presence of other people. The researcher in charge of the interviews held a Master's degree in Research in Social-Health Sciences at the time of the study. The researcher was not familiar with the subjects who participated in the study.

The main questions included in the script were: What did you feel when you found out you were going to receive a stoma? What beliefs did you have about the impact the stoma would have in your life? (what feelings did you have, what did you think); What feelings did you have when you saw the stoma for the first time?; Until today, what has the creation of the stoma cost you? The script was completed with questions related to the diversification criteria and sociodemographic variables. One interview was held per informant as a data saturation procedure was chosen through the progressive inclusion of new subjects in the study.

In the data analysis, a sequential scheme was adopted, based on the constant comparison of the data and the progressive incorporation of new informants until the saturation point of the categories was reached. The order of the analysis and the incorporation of subjects in the study was 1-4-9-8, until reaching the saturation point. An initial analysis was elaborated with one informant to establish a first code scheme; next, four interviews were held and analyzed to establish a second scheme; then, nine additional interviews were held to consolidate the findings and, finally, eight more interviews took place to saturate the information.

The procedure involved three phases: 1. transcription of the interviews and incorporation of the field notes; 2. inductive coding of the interviews, producing a descriptive code scheme in each dimension; grouping of the codes in broad categories; 3. interpretation with detailed readings of the data, constantly comparing the codes and formulating proposals that described the properties and dimensions of each code. The software Atlas-ti was used.

A triangular three-phase analysis was undertaken: in the first phase, the same researcher who held the interviews became responsible for the coding and, together with two other researchers (primary author and last author), they developed an initial interpretation of the findings. In a second phase, the results were presented during a meeting with the three stomal therapy nurses who had contacted the informants, with a view to contrasting the findings obtained from their clinical experience. In the final phase, the findings were again reviewed by the research team, which included the researchers from the first phase and the other authors of this study.

Written consent was requested from each participant, informing on the main objective and the conditions for participation, clarifying the voluntary nature of the participation. In addition, permission was requested to record the interviews. Each interview received a code, consisting of an initial that referred to the stomal therapy nurse who had made contact, followed by a number indicating the order of the interview in that nurse's population. The confidentiality of the information was guaranteed based on the legislation in force in Spain (Organic Law 15/1999 from December 13^th^ on the Protection of Personal Data). Authorization for the study was obtained from the Research Committee at the School of Nursing, Physiotherapy, Podiatry and Occupational Therapy of the Universidad de Málaga.

## Results

**Figure 1:**
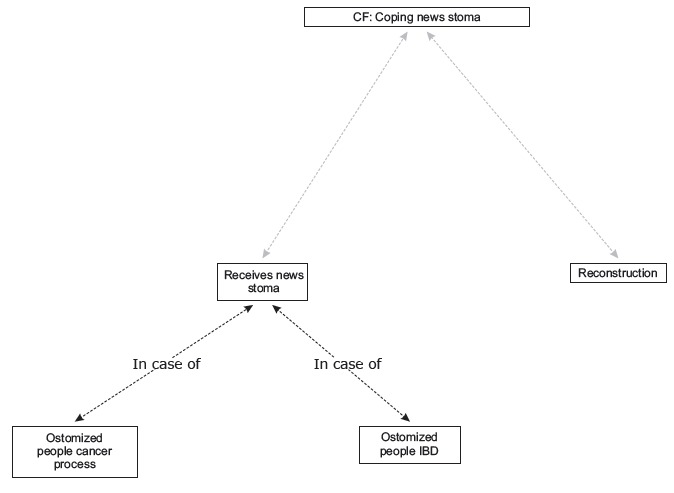
Network category: *Coping with the news about receiving a stoma" and "Meaning and experience of the new bodily reality*. Granada and Málaga, Spain, 2011

**Figure 2:**
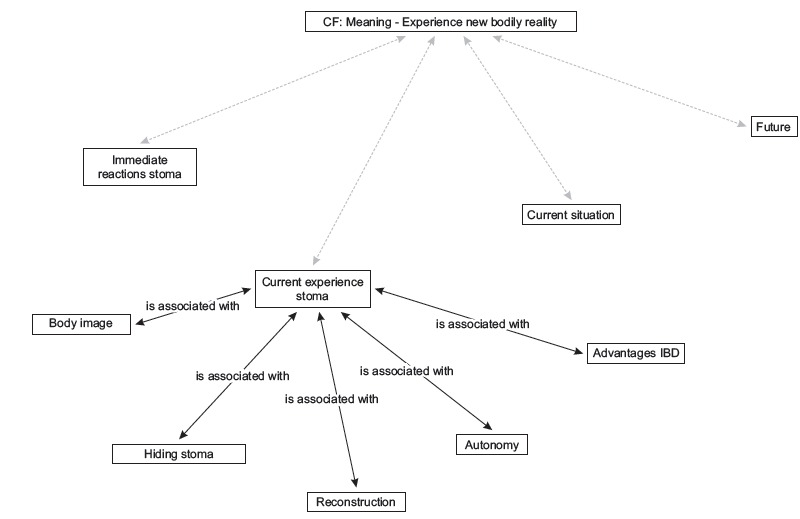
Network category: *Meaning - Experience new bodily reality*. Granada and Málaga, Spain, 2011

Two categories were obtained: "Coping with the news about receiving a stoma", with four codes distributed between two coding levels (Figure 1) and "Meaning and experience of the new bodily reality", with nine codes and two coding levels (Figure 2).

### Coping with the news about receiving a stoma

#### Receives news stoma

**Figure 3:**
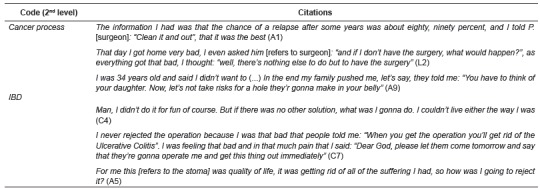
Citations Code: "Receives news stoma". Granada and Málaga, Spain, 2011

In this code, differences were found in the discourse of people ostomized due to a cancer process or IBD (Figure 3).

In cancer patients, the first reaction that predominates is rejection. In some cases, after receiving information on the different alternatives for the surgery, the prognosis etc., patients shift from rejection to acceptance when they realize that it is the best option to avoid future complications. Having this information allows them to participate in the decision process on the surgery. In other cases, the ostomy is rejected as long as possible, until the situation gets that bad that it is considered the final way out to move on with one's life.

Stoma patients due to IBD generally see the ostomy as the way to improve their quality of life, as something that will allow them to put an end to the suffering.

#### Reconstruction

When the stoma is temporary, it is observed that this factor contributes to better cope with the situation. In the informants' answers, they do not mind returning to the surgery room, nor the time it takes until the operation, because they know that sooner or later their current situation will be reverted.

*I got an emergency surgery and they installed a temporary ileostomy, supposedly as a quick fix because it was bad. They told me that, after some months, they would put things back together and so, which was what I wanted* (C5).

*I am more conformed because I know that, within a month or two, things will get back to normal. That's really what makes me more conformed. If it were permanent I would definitely be more down* (L2).

Nevertheless, it is also observed how, in some cases, despite counting on this possibility, people prefer to keep their stoma, indicating that it is doing well, that they have adapted to their new situation. They highlight that, if they have no guarantees for the results of the intervention, they prefer to stay as they are.

*Yes, it is now irreversible. There was a possibility but the surgeon told me: "you can get by for some time, but guarantees?" [...] In view of my current situation, if the doctor cannot give me these guarantees, I prefer to stay as I am. Today my quality of life is the way it was, and what is new* [refers to the stoma] *has already become part of me* (A2).

*When I want I can return, but I have to think, because if I get a new surgery, I'll have to go to the bathroom at least ten times per day for three years and I think that must be terrible. I am not upset, I have no inconveniences of any kind* (C1).

### Meaning and experience of a new bodily reality

After the stoma is in place, a recurring question is "will I be able to live as I used to?" The subjects perceive this new situation they are confronted with discouraged and afraid. Having background experience with an ostomized relative is a conditioning factor, observing that, in some cases, it exerted negative influence, but the opposite effect in others.

*If you're not hard it's difficult to accept, because it's not the surgery, it's everything it entails afterwards, because this one is for life, it's not finite, it's infinite, until you die. I was afraid, I used to think: "I cannot live as I used to anymore"* (A1)*.*

*I am already used to it because my mother has gone through this and my daughter too and my daughter-in-law. No, I haven't been concerned with this* [refers to the stoma] (L3).

*It was when I found out that the stoma was definitive that I broke down. I remembered my grandmother, she was eighty years old when she got hers. It escaped, it was always dirty, so I experienced it as a drama* (C3).

#### Immediate reactions to the stoma

Some people want to postpone the moment of seeing the stoma for the first time. This moment causes different feelings, including fear, impression, estrangement or curiosity.

*At first, I even rejected somewhat seeing it* (A2).

*The second day, my daughter told me: Mom, do you want to see it, how the pouch was transparent, and I said: No, no, let it for now. We'll see it afterwards* [smiles] (A4).

*The first reaction when I saw the stoma was curiosity, nothing else. C., who is very well prepared* [refers to the stomal therapy nurse]*, put a small mirror here, and there was nothing about seeing it. It's not pleasant, but well* (C6).

Some express their concern and fear about the self-care, in terms of not knowing how to treat the stoma or how to cope with the care for it. This concern particularly emerges when the patients have to leave the hospital, so that the insecurity emerges when they are no longer covered by the health fabric that has attended to their care needs thus far. They have to learn to manage their autonomy.

*What is true is that it was very scary to touch the stomas, and at first I thought I couldn't, that I wouldn't be able to* (L1).

*Taking care of the stoma myself, at first it looked like a cathedral to me* (C2).

#### Current experience with the stoma

As regards the altered body image, some people have already incorporated this change. They refer to the importance of seeing it as something normal, of accepting the stoma as part of them, even giving them names and linking them with adjectives.

*It's assuming that it's there and that's it. That you carry it there like your cell phone in your pocket, or a pack of gums* [laughs] (C4).

*You weren't a hole in your belly, you were much more, and that's why I tell you that I'm dealing with it very well [...] If I have to wear a bikini, I will. That is, it's not for nothing, but my stoma is very pretty, it's beautiful* [laughs] (A9).

*I look in the mirror and say: In fact, I'm sexy* [laughs]. *I call it: bum, tummy* [refers to the stoma. Laughs] (A3).

Others do not see it as something normal, they feel different. In these cases, they do not see the stoma as part of them, tending towards thingification.

*See your belly with this* [refers to the stoma] *[...] It was something totally new to me, something I didn't know. But what can I do if this happened* (L1).

Some of the concerns the informants express refer to their perception of how this bodily change could influence their finding of a partner or to the difficulties to get accustomed to this change that has taken place in their body.

*If I didn't have a partner, it would be much more difficult to find one. It's not the same to have an ileostomy or not, that's clear* (C5).

*My life has changed, as soon as I take my clothes of I see the change in me, but I increasingly feel more normal, more normalized in my new situation* (L2).

What the hiding of the stoma is concerned, the reactions are again variable. In the reports, it is observed how some informants do not see any problem in other people knowing about their situation. They consider that it is not something they have to hide, or something to be ashamed of. In addition, a trend is observed to naturalize the problem with the surrounding family.

*I don't have any problem, if I have to go to the beach I will and if the belt is visible I don't mind. I don't have anything to hide, what am I going to hide?* (C1).

*My daughter, she has always seen me since she was a little girl. She used to sit with me in the bathroom and pass the plugs, she helped me to cut them, So for her it's normal* (A9).

On the opposite, in other informants' discourse, there is concern with the fact that the others realize that they are wearing a stoma. They are afraid of feeling that they are being watched.

*I find myself and I know that I have it [refers to the pouch], and I don't know if the person who is seeing me will notice it or not* (C3).

Autonomy is another key issue in this code. The informants highlight the importance of not depending on anyone or anything to take care of the stoma.

*Even if a person wants to help you, you also have to try not to depend on her, but to, if you can, be self-sufficient* (A2).

#### Other considerations on the current situation

Normality rules in the informants' discourse when they refer to their current life, as the fact that they accept and adapt themselves to the digestive stoma makes it easier for people to keep on doing almost everything they used to before the intervention, as well as to maintain they plans they had for their life before the problem happened.

In some informants' discourse, this perceived normality even reaches the point of forgetting that they have a stoma on some occasions or of no longer finding the fact important.

*I have accepted it, in fact, I often forget that I have a pouch* (C1).

*At first, I found it very important, everything revolved around the stoma, but now I sometimes even forget* (C3).

#### Future

Aging or being in a situation of disability that prevents them from taking charge of their stoma are some of the concerns the informants manifest when they are asked about their future.

*I only ask that I will not fall short of my hands and sight. That is fundamental to be able to keep on taking care of the stoma, without depending on anyone, to maintain my autonomy* (C8).

For some ostomized patients due to an oncological process, the uncertainty of not knowing whether the disease will reproduce itself or not is also at stake.

*I still go in circles around the theme Cancer. It's something I believe I have under control, but it's still there, I still have the nightmares* (C9).

## Discussion

Among the study findings, the difference between the subjects whose disease that caused the stoma is cancer and the subjects with IBD should be highlighted. In the first case, the results show that the initial impact of the news supposes a rejection of the surgery, in contrast with the apparent normality of the second group's reaction, as part of the natural cycle of their therapeutic procedure.

When analyzing the answers of the people diagnosed with cancer in further depth, one might think that the fact of receiving the news about the diagnosis has a lesser impact than knowing that they will receive a stoma, considering the change in the bodily integrity as more threatening than the actual threat to health this diagnosis entails. Some authors indicate that physical deformities are an important source of stigma in society, which is why the fear of stigma partially explains this finding^15^. On the other hand, cancer is a disease associated with death, so that installing the stoma could intensify the perceived severity associated with the disease even further, being an invasive procedure with clear physiological and psychological consequences. These two aspects, fear of stigmatization and worsening of the perceived severity, could justify the informants' rejection of the ostomy, as if it were an attitude of avoiding the disease and the violation of their body image, accepting it only when the severity of the disease requires so, in line with other studies^8^. In this sense, the family's role is also relevant, which in some of the situations described acts as a conditioning factor for the patient to make the decision to move on with the intervention. The relevant role of the family has been demonstrated to help the ostomized subject with his social reinsertion^16^. In addition, the worsening in the family's quality of life is observed due to the whole monitoring process^17^.

On the opposite, in the case of IBD patients, the therapeutic option of the ostomy is associated with a better quality of life. Some studies focus on this fact, concluding that, for IBD patients, the surgery is a resource needed to facilitate the symptom management and improve the quality of life^4^. 

The results also highlight the importance of the person's participation in the decision process when the installation of the stoma is proposed, guaranteeing the principle of autonomy. The decision making is directly conditioned by the information the patient receives. In that sense, some studies conclude that, when the subject is informed about the need for the intervention, the impact is greater when he does not know what procedure he will be submitted to^18^. 

The other central category analyzed in this study is related to the meanings and experiences the subjects manifest in view of the modification of their body after the surgery. After the surgery, the stoma patients need to cope with a new situation. Something has changed, they are not like they used to be, they have something additional they need to incorporate to reconstruct their body image. Some authors call this situation the "gain-loss dichotomy"^19^. Ostomized people do not only lose part of their body (intestinal segment), with the consequent change in their body image, in their daily routine, as well as the loss of continence, trust etc. This also implies the incorporation of a range of elements: the stoma, the pouch and the feeling of being different^12^.

One conflicting point emerges when the subjects need to face the self-care at home, where the fear and concern again appear when they do not know how to treat the stoma. The return home is supposedly one of the most complicated periods in this process^20^.

Some authors affirm that the patients manifest shame when they perceive that they are carrying an unwanted attribute, considering hiding as a way to disguise it^15^. This situation was also described in part of the informants included in this study, who are concerned with the fact that other people might notice that they are carrying a stoma. These subjects tend to preserve control of the information about themselves, so as not to feel excluded, which is called the "Cinderella complex"^15^. On the opposite, a discourse against this situation is also predominant. Some subjects have accepted the bodily change and have reconciled it with themselves as well as with society. How long the patients have had the stoma may be an explanatory factor of this variation and of other situations described, which is an aspect to be considered in further depth in future research.

This process of rediscovering a modified body can be analyzed from a phenomenological perspective as a process of resignifying the body, considered as a vehicle through which people understand and experience their world^21^. This concept is important for clinical practice, as the health services should offer resources that help this type of patients to get reconciled with a life that has undergone a radical change, as well as to align the patients' expectations of what life with a whether temporary^22^ or indwelling^23^ digestive stoma can be like. In that sense, scientific evidence shows that structured educational programs for ostomized patients significantly improve their health-related quality of life^24^.

The nursing professionals play a fundamental role in these patients' self-care^25^. In this respect, the stomal therapy nurse, as a model of Advanced Nursing Practice, is an effective alternative to develop both this type of cognitive-behavioral resources and to promote the patients' autonomy in everything related to care for the stoma.

What the study limitations are concerned, qualitative research permits inquiries in concrete contexts, incorporating the specific social situation in that context. In this case, the study was undertaken with a Spanish population, which is novel in view of the lack of studies on the experiences of stoma patients in that country. Based on the discussion, it can be concluded that the findings can be transferred, in view of the results and contexts of other studies, although further research is needed to analyze the social variants that may exert influence.

## Conclusion

The coping with the news about the stoma is conditioned by the type of disease that originated it. It is more ineffective for cancer patients. After the ostomy, the normalization of the process is the trend observed in most of the informants interviewed. The nursing professionals play a fundamental role for the normalization of the process, so that their activities should include the implementation of cognitive and behavioral reinforcement interventions, besides the encouragement of the digestive stoma patients' self-care and autonomy.

## References

[1] Siegel R, DeSantis C, Jemal A (2014). Colorectal cancer statistics, 2014. Cancer J Clin.

[2] Danielsen AK, Soerensen EE, Burcharth K, Rosenberg J (2013). Learning to live with a permanent intestinal ostomy impact on everyday life and educational needs. J Wound Ostomy Continence Nurs.

[3] Danielsen AK (2013). Life after stoma creation. Dan Med J.

[4] Pereira APS, Cesarino CB, Martins MRI, Pinto MH, Netinho JG (2012). Associations among socio-demographic and clinical factors and the quality of life of ostomized patients Rev. Latino-Am. Enfermagem.

[5] Zhang JE, Wong FK, You LM, Zheng MC, Li Q, Zhang BY (2013). Effects of enterostomal nurse telephone follow-up on postoperative adjustment of discharged colostomy patients. Cancer Nurs.

[6] Tao H, Songwathana P, Isaramalai S, Zhang Y (2014). Personal awareness and behavioural choices on having a stoma a qualitative metasynthesis. J Clin Nurse.

[7] Kenderian S, Stephens EK, Jatoi A (2014). Ostomies in rectal cancer patients what is their psychosocial impact?. Eur J Cancer. Care. (Engl).

[8] Barnabe NC, Dell Acqua MCQ (2008). Coping strategies of ostomized individuals Rev. Latino-Am. Enfermagem.

[9] Lazarus RS (2000). Estrés y emoción: manejo e implicaciones en nuestra salud.

[10] Popek S, Grant M, Gemmill R, Wendel CS, Mohler MJ, Rawl SM (2010). Overcoming challenges life with an ostomy. Am J Surg.

[11] Wilson TR, Birks YF, Alexander DJ (2010). A qualitative study of patient perspectives of health-related quality of life in colorectal cancer comparison with disease-specific evaluation tools. Colorectal Dis.

[12] Thorpe G, McArthur M, Richardson B (2009). Bodily change following faecal stoma formation qualitative interpretive synthesis. J Adv Nurs.

[13] Li CC (2009). Sexuality among patients with a colostomy an exploration of the influences of gender, sexual orientation, and Asian heritage. J Wound Ostomy Continence Nurs.

[14] Andrade C, Holanda A (2010). Apontamentos sobre pesquisa qualitativa e pesquisa empírico-fenomenológica. Estud Psicol. (Campinas).

[15] Goffman E (2008). Estigma. La identidad deteriorada.

[16] Menezes APS, Quintana JF (2008). A percepção do indivíduo estomizado quanto à sua situação. RBPS.

[17] Palma E, Simonetti V, Franchelli P, Pavone D, Cicolini G (2012). An observational study of family caregivers' quality of life caring for patients with a stoma. Gastroenterol Nurs.

[18] De Brum CN, Sodré BS, Prevedello PV, Quinhones SWM (2010). O processo de viver dos pacientes adultos com ostomias permanentes uma revisão de literatura. Rev Pesqui: Cuid. Fundam.

[19] Santos VLCG, Sawaia BB (2000). The pouch mediating the relation between "being an ostomized person" and "being professional" analysis of a pedagogic strategy. Rev. Latino-Am. Enfermagem.

[20] Richbourg L, Torpe JM, Rapp CJ (2007). Difficulties Exerienced by the ostomates after hospital discharge. J Wound Ostomy Continence Nurs.

[21] Merleau-Ponty M (1975). Fenomenología de la percepción.

[22] Neuman HB, Park J, Fuzesi S, Temple LK (2012). Rectal cancer patients' quality of life with a temporary stoma shifting perspectives. Dis Colon Rectum.

[23] Cheng F, Meng AF, Yang LF, Zhang YN (2013). The correlation between ostomy knowledge and self-care ability with psychosocial adjustment in Chinese patients with a permanent colostomy: a descriptive study. Ostomy Wound Manage.

[24] Danielsen AK, Rosenberg J (2014). Health related quality of life may increase when patients with a stoma attend patient education--a case-control study. PLoS ONE.

[25] Mota MS, Gomes GC, Petuco VM, Heck RM, Barros EJ, Gomes VL (2015). Facilitators of the transition process for the self-care of the person with stoma subsidies for Nursing. Rev Esc Enferm USP.

